# Activation of the NLRP3 Inflammasome Increases the IL-1β Level and Decreases GLUT4 Translocation in Skeletal Muscle during Insulin Resistance

**DOI:** 10.3390/ijms221910212

**Published:** 2021-09-23

**Authors:** Luan Américo-Da-Silva, Javiera Aguilera, Oscar Quinteros-Waltemath, Pablo Sánchez-Aguilera, Javier Russell, Cynthia Cadagan, Roberto Meneses-Valdés, Gina Sánchez, Manuel Estrada, Gonzalo Jorquera, Genaro Barrientos, Paola Llanos

**Affiliations:** 1 Instituto de Investigación en Ciencias Odontológicas, Facultad de Odontología, Universidad de Chile, Santiago 8380544, Chile; ldasilva@odontologia.uchile.cl (L.A.-D.-S.); javiera.aguilera.m@gmail.com (J.A.); oscar.qw@gmail.com (O.Q.-W.); cynthia.cadagan@ug.uchile.cl (C.C.); 2 Centro de Estudios en Ejercicio, Metabolismo y Cáncer, Facultad de Medicina, Universidad de Chile, Santiago 8380453, Chile; pabloskine@gmail.com (P.S.-A.); roberto.meneses@ug.uchile.cl (R.M.-V.); 3 Escuela de Pedagogía en Educación Física, Facultad de Educación, Universidad Autónoma de Chile, Santiago 8900000, Chile; javier.russell@uautonoma.cl; 4 Programa de Fisiopatología, ICBM, Facultad de Medicina, Universidad de Chile, Santiago 8380453, Chile; gisanchez@uchile.cl; 5 Programa de Fisiología y Biofísica, ICBM, Facultad de Medicina, Universidad de Chile, Santiago 8380453, Chile; iestrada@med.uchile.cl; 6 Centro de Neurobiología y Fisiopatología Integrativa (CENFI), Instituto de Fisiología, Facultad de Ciencias, Universidad de Valparaíso, Valparaíso 2360102, Chile; gonzalo.jorquera@uv.cl

**Keywords:** high-fat diet, myokines, caspase-1, GSDMD, NALP3, inflammation, GLUT4

## Abstract

Low-grade chronic inflammation plays a pivotal role in the pathogenesis of insulin resistance (IR), and skeletal muscle has a central role in this condition. NLRP3 inflammasome activation pathways promote low-grade chronic inflammation in several tissues. However, a direct link between IR and NLRP3 inflammasome activation in skeletal muscle has not been reported. Here, we evaluated the NLRP3 inflammasome components and their role in GLUT4 translocation impairment in skeletal muscle during IR. Male C57BL/6J mice were fed with a normal control diet (NCD) or high-fat diet (HFD) for 8 weeks. The protein levels of NLRP3, ASC, caspase-1, gasdermin-D (GSDMD), and interleukin (IL)-1β were measured in both homogenized and isolated fibers from the flexor digitorum brevis (FDB) or soleus muscle. GLUT4 translocation was determined through GLUT4*myc*-eGFP electroporation of the FBD muscle. Our results, obtained using immunofluorescence, showed that adult skeletal muscle expresses the inflammasome components. In the FDB and soleus muscles, homogenates from HFD-fed mice, we found increased protein levels of NLRP3 and ASC, higher activation of caspase-1, and elevated IL-1β in its mature form, compared to NCD-fed mice. Moreover, GSDMD, a protein that mediates IL-1β secretion, was found to be increased in HFD-fed-mice muscles. Interestingly, MCC950, a specific pharmacological NLRP3 inflammasome inhibitor, promoted GLUT4 translocation in fibers isolated from the FDB muscle of NCD- and HFD-fed mice. In conclusion, we found increased NLRP3 inflammasome components in adult skeletal muscle of obese insulin-resistant animals, which might contribute to the low-grade chronic metabolic inflammation of skeletal muscle and IR development.

## 1. Introduction

According to the World Health Organization, in 2016, 1.9 billion people were overweight and 650 million were obese, and it is projected that in 2040 there will be ~642 million people with type 2 diabetes [1]. These conditions are caused by the overconsumption of caloric-dense food with a high fat and refined sugar content. Both insulin resistance (IR) and obesity are associated with low-grade chronic inflammation [1]. IR is characterized by a reduced response to insulin, frequently associated with increased levels of circulating hormones, metabolic inflexibility, and impaired glucose uptake [2]. A hallmark of being overweight and obesity is the development of insulin resistance in the skeletal muscle, which is the main site of post-prandial insulin-dependent glucose uptake, and this process requires GLUT4 translocation [3]. In obesity, there is an expansion of fat tissue and increased release of pro-inflammatory mediators, including IL-1β, which have been proposed to impair insulin action [4]. The skeletal muscle represents around 40% of the weight of an adult individual, and it has been recognized as an endocrine organ which produces and releases several myokines [5,6]. Recently, emerging evidence has suggested that muscle secretion of inflammatory mediators could be associated with IR development [7]. 

The mechanisms involved in the occurrence of chronic low-grade inflammation during obesity-associated insulin resistance in the absence of an overt acute infection or a well-defined autoimmune process remain unknown [8]. The cytoplasmic nucleotide-binding oligomerization domain-like receptor family (NOD-like) pyrin domain containing 3 (NLRP3) inflammasome has been implicated in the development of inflammation and insulin resistance in diverse tissues [9]. The NLRP3 inflammasome is a multi-protein complex that recruits pro-caspase-1 via ASC proteins and directly activates caspase-1, the enzyme responsible for the transformation of pro-IL-1β into mature IL-1β [9,10]. Activation of the NLRP3 inflammasome has been extensively studied in macrophages [11], and its function requires two steps, known as priming and activation [9]. The priming step is provided by inflammatory stimuli, such as TLR4 agonists, which stimulate NF-κB-mediated NLRP3 and pro-IL-1β gene expression, and the activation step is triggered by a variety of priming stimuli by a pathogen (PAMPs) and danger-associated molecular patterns (DAMPs), thereby promoting NLRP3 inflammasome assembly and caspase-1-mediated IL-1β secretion [9]. The IL-1β protein lacks the signal peptide required for secretion through the classic secretory pathway [12], and it has been suggested that it is released to the extracellular milieu by unconventional mechanisms, such as gasdermin D (GSDMD)-mediated secretion [13]. It has been proposed that after proteolytic activation via caspase-1 activity, GSDMD N-terminal domains insert themselves into the plasma membrane, promoting the formation of oligomeric pores, which allow IL-1β release to the extracellular media [14]. However, the role of the NLRP3 inflammasome in IL-1β release through GSDMD pores during obesity-induced IR in skeletal muscle tissue remains unexplored.

In this work, we address the hypothesis that during obesity-induced IR, there is an increase in the NLRP3 inflammasome complex, IL-1β, and GSDMD protein content in the skeletal muscle. We propose that in skeletal muscle, obesity activates the NLRP3 inflammasome, which in turn cleaves and activates IL-1β. We study the possibility that NLRP3 activity from the skeletal muscle could impair the insulin-dependent GLUT4 translocation in this tissue. To address this hypothesis, we used a well-characterized murine model of obesity-induced IR by feeding mice with a high-fat diet (HFD) for 8 weeks. We measured mRNA, protein levels, and cellular location of NLRP3, GSDMD, and IL-1β in the skeletal muscle of obese and control animals. We also tested the effect of MCC950, a highly specific inhibitor of NLRP3, on insulin resistance in the skeletal muscle. Based on our results, we suggest that the NLRP3 inflammasome participates in the pathogenesis of obesity-induced IR in skeletal muscle tissue.

## 2. Results

### 2.1. HFD Promotes an IL-1β Plasma Level Increase in Mice 

**Figure 1:**
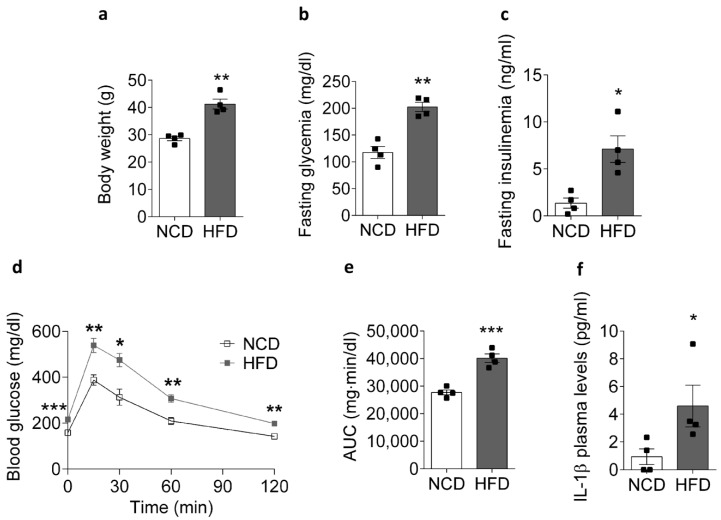
Physiological and metabolic parameters of NCD- and HFD-fed mice. Comparison of (**a**) body weight, (**b**) fasting blood glucose, (**c**) plasma insulin, (**d**) intraperitoneal glucose tolerance test, (**e**) area under the glucose curve, and (**f**) IL-1β in mice fed for 8 weeks with an NCD or HFD. Data are shown as the mean ± SEM. * *p* < 0.05 (*n* = 4), ** *p* < 0.01, and *** *p* < 0.001 vs. HFD. NCD, normal control diet; HFD, high-fat diet.

To confirm that chronic exposure to a HFD for 8 weeks would promote insulin resistance in our animal model, we measured body weight, glycemia, and plasma insulin in obese and control mice (Figure 1). HFD-fed mice showed a marked weight gain compared to NCD-fed animals, reaching an average body weight of 41.2 ± 1.8 and 28.6 ± 0.8 g, respectively (Figure 1a). In addition, the fasting plasma glucose level (Figure 1b) and serum insulin (Figure 1c) were significantly higher in the HFD-fed mice compared to the control mice. Consistent with the above data, we obtained an impaired intraperitoneal glucose tolerance test (IPGTT) (Figure 1d) with an increase in the calculated area under the curve (AUC) of the IPGTT plot in the HFD-fed mice relative to the control mice, with values of 27,705 ± 899 and 40,146 ± 1561 mg min/dl, respectively (Figure 1e). Together, these results confirm that chronic feeding with a HFD caused insulin resistance in our mice. 

Recently, it has been reported that obesity-associated chronic low-grade inflammation is responsible for the decrease in insulin sensitivity [15]. Interestingly, the pro-inflammatory cytokine IL-1β was increased in the plasma of the HFD-fed mice compared to the NCD-fed mice, reaching 4.6 ± 1.5 and 0.9 ± 0.6 pg/mL, respectively (Figure 1f). Taken together, these findings suggest that insulin resistance and chronic low-grade inflammation are associated with a reduction in insulin sensitivity and increased levels of IL-1β. 

### 2.2. HFD Is Associated with Increased NLRP3 mRNA Levels in Mice 

**Figure 2:**
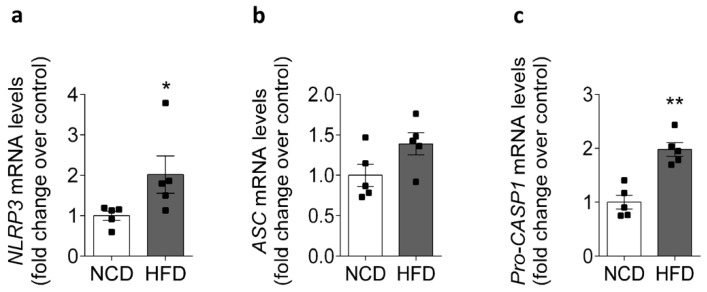
Expression of the NLRP3 inflammasome in the FDB muscle from NCD- and HFD-fed mice. FDB muscle was collected from NCD- or HFD-fed mice, and the mRNA level of the NLRP3 inflammasome was assessed by RT-qPCR and normalized to housekeeping gene Rplp0. The comparison of mRNA levels of (**a**) NLRP3, (**b**) ASC, and (**c**) pro-caspase-1. The expression of ASC did not change significantly after 8 weeks of an HFD. Data are shown as the mean ± SEM (*n* = 5). * *p* < 0.05 and ** *p* < 0.01 vs. HFD, determined by Student’s *t*-test statistical analysis. NCD, normal control diet; HFD, high-fat diet.

Skeletal muscle is not just a component of the locomotor system, but is also a tissue with a high metabolic rate, and it is the main tissue that is responsive to insulin-stimulated glucose transport [3]. Mounting evidence supports the role of skeletal muscle as a secretory organ, releasing several myokines, including IL-1β [7,16]. IL-1β is produced in an inactive pro-form that requires cleavage by the NLRP3 inflammasome to be released from the cell, and for its subsequent biological activity [9]. In order to evaluate whether HFD modifies the NLRP3 inflammasome component content in skeletal muscle, we performed an RT-qPCR assay for NLRP3, ASC, and pro-caspase-1 in homogenates of the FDB muscle from NCD- or HFD-fed mice. As shown in Figure 2a,c, compared with the control group, the skeletal muscle had significantly higher relative mRNA expression levels of NLRP3 (1.0 ± 0.11- and 2.0 ± 0.46-fold) and pro-caspase-1 (1.0 ± 0.13- and 2.0 ± 0.13-fold), while for ASC no visible change was observed (1.0 ± 0.14- and 1.4 ± 0.14-fold, Figure 2b). 

### 2.3. Increased NLRP3 Inflammasome Components in Skeletal Muscle Isolated from HFD-Fed Mice 

**Figure 3:**
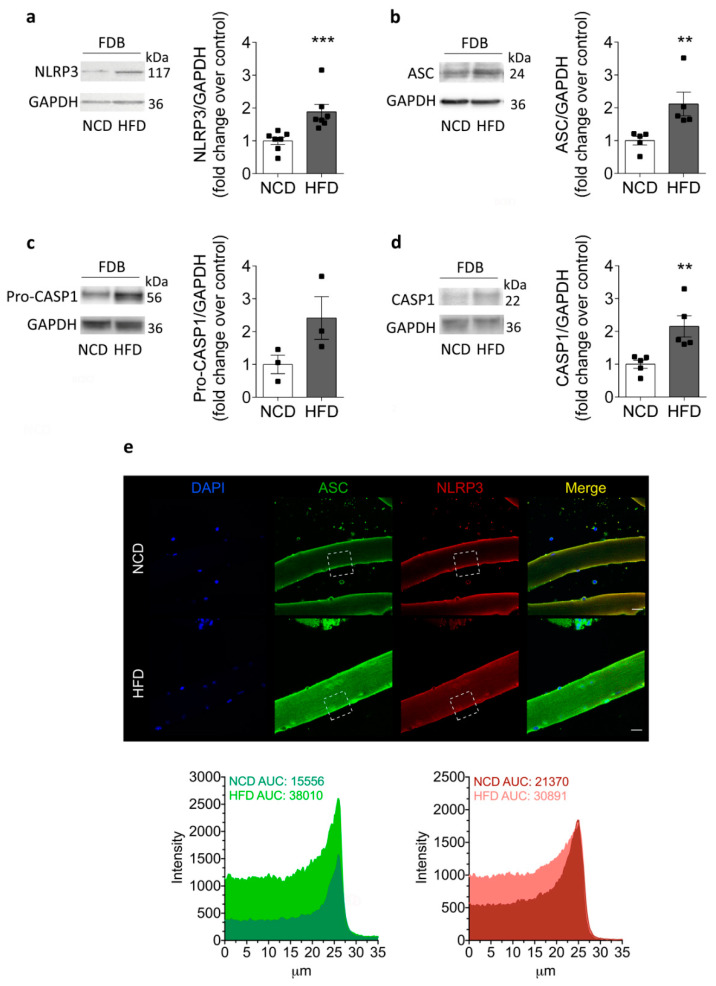
NLRP3 inflammasome protein content and localization in the skeletal muscle from HFD compared to NCD-fed mice. Representative Western blot and quantification showing (**a**) NLRP3, (**b**) ASC, (**c**) pro-caspase-1, and (**d**) caspase-1 protein content in homogenates. The samples were prepared from FDB muscle isolated from NCD- or HFD-fed mice after 8 weeks of an HFD. GAPDH was used as a housekeeping loading control. Each lane was loaded with 60 μg of total protein. Values represent the mean ± SEM (*n* = 3–7). ** *p* < 0.01 and *** *p* < 0.001, determined by Student’s *t*-test. (**e**) Representative confocal images of isolated adult FDB fibers from NCD- and HFD-fed mice illustrate the localization of ASC (green) and NLRP3 (red). DAPI staining of the nuclei is shown in blue. The area under curve (AUC) was measured on white squares, and quantified as fluorescence intensity. Scale bar: 20 μm.

In concordance with the above results, the NLRP3 protein content was increased in HFD-fed mice compared to NCD-fed mice, as measured by Western blot in the FDB muscle (Figure 3a). The NLRP3 protein content reached values of 1.9 ± 0.2- and 1.0 ± 0.1-fold in HFD- and NCD-fed mice, respectively. In addition, our results showed that ASC and procapasase-1 increased their immunoreactive band intensities (2.1 ± 0.4- and 2.4 ± 0.6-fold) in HFD-fed mice compared to NCD-fed mice (1.0 ± 0.1- and 1.0 ± 0.3-fold), respectively (Figure 3b,c). Active caspase-1 also increased from 1.0 ± 0.1 in NCD-fed mice to 2.1 ± 0.3 in HFD-fed mice (Figure 3d). Since the FDB is a muscle composed mainly of fast, glycolytic skeletal fibers, we evaluated whether the components of the inflammasome were also modified in the soleus muscle, which is mainly a slow, oxidative skeletal muscle. Interestingly, similar results were obtained in soleus muscle homogenates, suggesting that augmented NLRP3 protein content is a phenomenon that occurs in different types of muscles during insulin resistance (Supplementary Figure S1). In addition, we analyzed the localization of NLRP3 inflammasome components in isolated skeletal muscle fibers (Figure 3e). Immunofluorescence detection of the NLRP3 and ASC proteins showed a sarcoplasmic localization, with increased fluorescence intensity in fibers isolated from HFD animals compared to controls. Together, our findings suggest that skeletal muscle could contribute to plasma IL-1β through increased NLRP3 expression in this tissue during insulin resistance. 

### 2.4. IL-1β Increases in the Skeletal Muscle of HFD-Fed Mice

**Figure 4:**
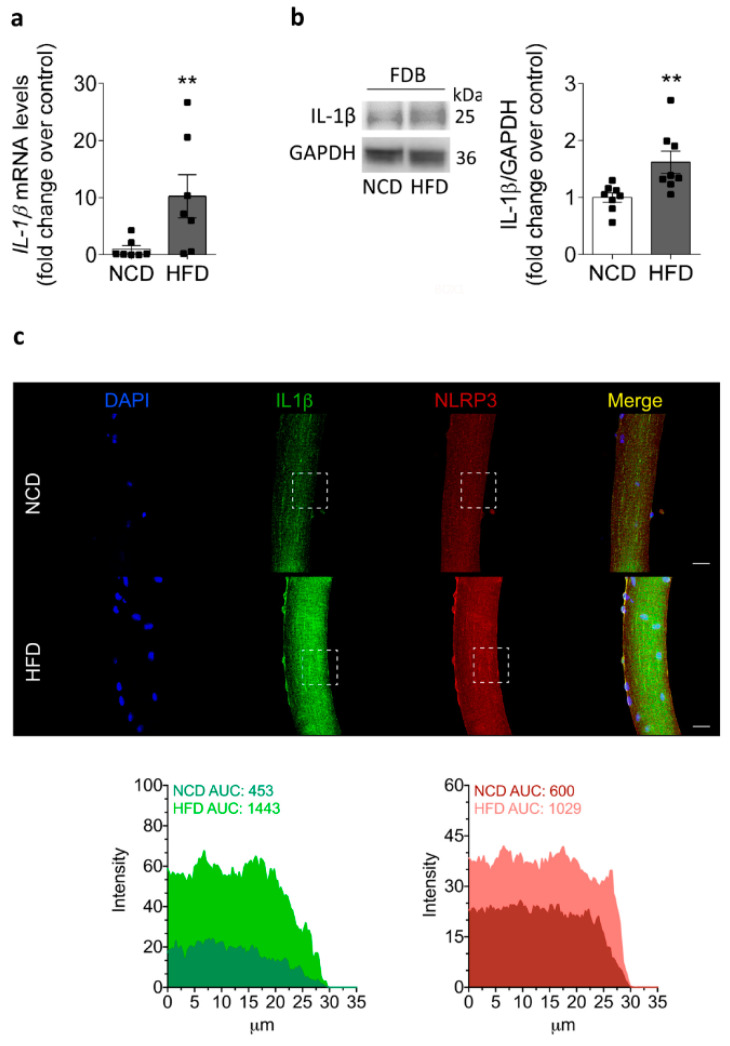
Increased IL-1β mRNA, protein levels, and distribution in the skeletal muscle of HFD-fed mice. (**a**) Relative IL-1β mRNA levels in isolated adult FDB fibers from NCD- and HFD-fed mice measured by RT-qPCR and normalized to GAPDH. Values represent the mean ± SEM from six independent determinations. ** *p* < 0.01, determined by Student’s *t*-test statistical analysis. (**b**) Representative Western blot showing IL-1β protein content in homogenates prepared from the FDB muscle isolated from NCD- or HFD-fed mice at 8 weeks. Each lane was loaded with 60 μg of total protein. Values represent the mean ± SEM (*n* = 7). ** *p* < 0.01, determined by Student’s *t*-test. (**c**) Representative confocal images of isolated adult FDB fibers from NCD- and HFD-fed mice, illustrating the localization of IL-1β (green) and NLRP3 (red). DAPI staining of the nuclei is shown in blue. The area under the curve (AUC) was measured on white squares, and represented as fluorescence intensity. Scale bar: 20 μm.

The function of the NLRP3 inflammasome has been largely associated with the conversion of pro-IL-1β into the IL-1β mature form for secretion [9]. Therefore, we measured both IL-1β mRNA and protein content in the fiber culture or homogenate of FDB from NCD- and HFD-fed mice, respectively. As shown in Figure 4, HFD-fed mice exhibited an increase in IL-1β mRNA (10.2 ± 3.8-fold) compared to NCD-fed mice (1.0 ± 0.6-fold) from isolated skeletal muscle fibers (Figure 4a). Then, we evaluated IL-1β protein content by Western blot in HFD-fed mice after 8 weeks (Figure 4b). An immunoreactive band was detected in the homogenates of the FDB muscle. In concordance with the mRNA data, the IL-1β content was increased in HFD-fed mice compared to NCD-fed mice, reaching values of 1.6 ± 0.2- and 1.0 ± 0.1-fold, respectively. In addition, to determine the distribution of IL-1β and NLRP3 in skeletal muscle, we evaluated the IL-1β and NLRP3 staining in a single muscle fiber isolated from the FDB muscle from both NCD- and HFD-fed mice (Figure 4c). These experiments showed a striated cytosolic pattern for IL-1β, which was more intense in the isolated fiber from HFD mice compared to the fiber from control mice. Based on these results, we suggest that both NLRP3 inflammasome priming and assembly steps are activated in skeletal muscle during insulin resistance. 

### 2.5. Chronic HFD Feeding Increases the Levels of Active Caspase-1 and the GSDMD Content 

**Figure 5:**
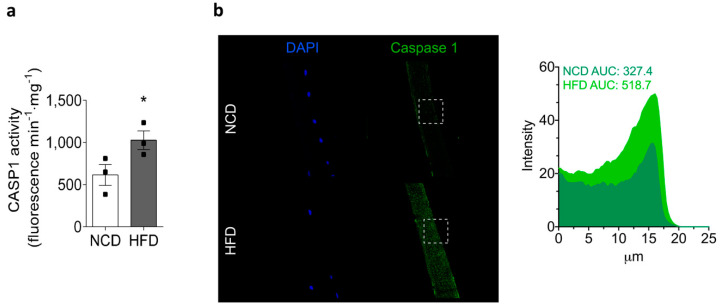
HFD increases caspase-1 activity in the skeletal muscle. Gastrocnemius muscles were collected from NCD- or HFD-fed mice, homogenized, and caspase-1 activity was measured. Values represent the mean ± SEM (*n* = 3). * *p* < 0.05, determined by Student’s *t*-test. (**a**) Caspase-1 activity in muscle lysates was measured with a caspase-1 fluorometric kit. (**b**) Representative confocal images of isolated adult FDB fibers from NCD- and HFD-fed mice illustrate the localization of caspase-1 (green). Nuclei were stained with DAPI (blue). The area under the curve (AUC) was measured on white squares, and represented as fluorescence intensity. Scale bar: 20 μm.

On stimulation, NLRP3 inflammasomes oligomerize to form large multimolecular complexes that control caspase-1 activity and the subsequent IL-1β processing [9]. As mentioned above, our results showed an increase in caspase-1 and IL-1β content in the skeletal muscle of HFD-fed mice (Figure 2 and Figure 3, respectively). Next, we assessed caspase-1 activity in homogenates of the skeletal muscle and its localization in isolated skeletal muscle fibers from both experimental groups (Figure 5). Our measurements showed that caspase-1 activity was significantly increased in HFD-fed mice, reaching values of 1028 ± 110 compared to 615 ± 124 fluorescence min^−1^·mg^−1^ in NCD-fed mice (Figure 5a). We measured caspase-1 activity in the gastrocnemius muscle, which is a mixed muscle, as its size allowed us to collect enough protein to perform the assay. As with NLRP3 inflammasome components, caspase-1-specific staining was localized in the cytoplasm, and the signal was increased in FDB skeletal muscle fibers isolated from HFD-fed mice relative to NCD-fed mice (Figure 5b). 

**Figure 6:**
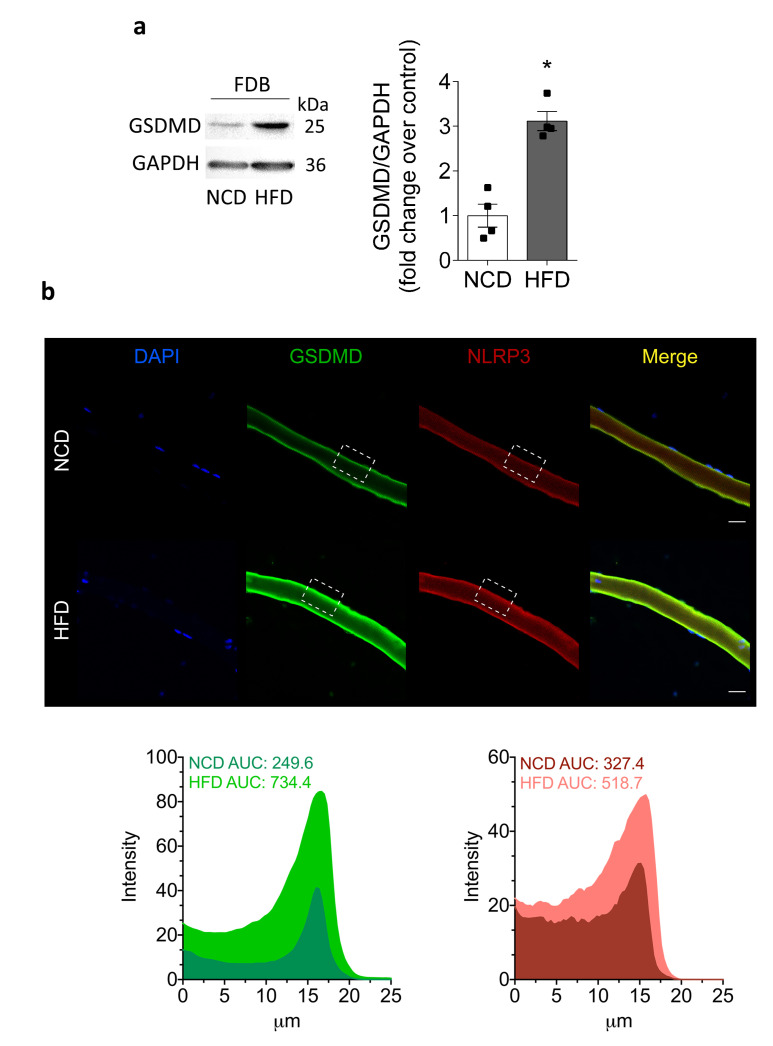
Cleaved GSDMD increases in HFD-fed mice. We dissected and homogenized FDB muscles from NCD- or HFD-fed mice and assessed the GSDMD level by Western blot. We also studied GSDMD subcellular location in fixed isolated fibers by immunofluorescence. (**a**) Representative Western blot and quantification showing cleaved GSDMD; 60 µg of lysate was loaded in each lane. The GSDMD bands were observed at 25 kDa. Values represent the mean ± SEM (*n* = 4). * *p* < 0.05, determined by Student’s *t*-test. (**b**) Representative confocal images of isolated adult FDB fibers from NCD- and HFD-fed mice illustrating the localization of GSDMD (green) and NLRP3 (red). DAPI staining of the nuclei is shown in blue. The area under the curve (AUC) was measured on white squares, and represented as fluorescence intensity. Scale bar: 20 μm.

Recently, it has been proposed that the GSDMD protein forms oligomeric pores that mediate IL-1β release, and it has been identified as a new target of NLRP3 inflammasome activation in different cell types, including macrophages [17]. In order to assess the GSDMD level and localization, we performed Western blot and immunofluorescence imaging in the skeletal muscle. Of note, GSDMD increased by 3.1 ± 0.2 in FDB muscle from HFD-fed mice, compared to 1.0 ± 0.3-fold in NCD-fed mice (Figure 6a). Furthermore, our immunofluorescence confocal images confirmed an increased level of GSDMD in single muscle fibers isolated from HFD-fed mice (Figure 6b). It should be noted that GSDMD showed a peripheral distribution, suggesting that GSDMD could oligomerize in the sarcolemma to form pores that mediate IL-1β release from the skeletal muscle of HFD-fed mice. 

### 2.6. Insulin-Dependent GLUT4 Translocation Is Improved by MCC950 

**Figure 7:**
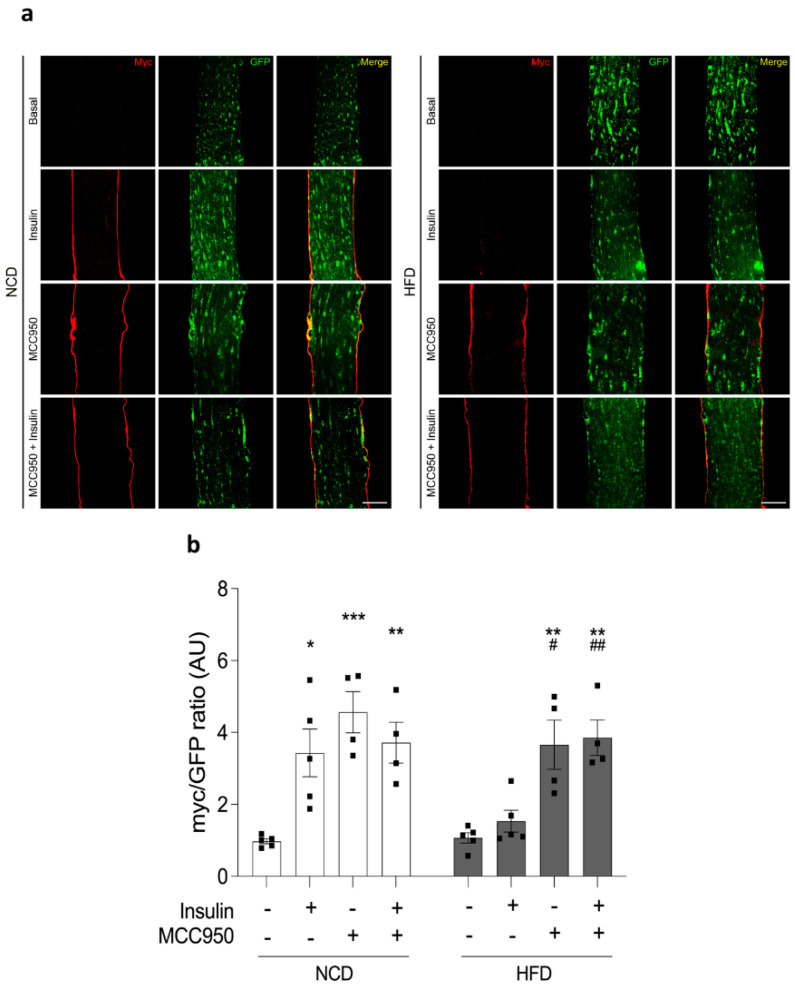
MCC950 improves the insulin-mediated GLUT4*myc*-eGFP translocation to the cell surface. (**a**) Representative images showing GLUT4 distribution in fibers electroporated with the GLUT4*myc*-eGFP (green) chimera plasmids. The fibers were isolated from NCD- or HFD-fed mice. The extracellular *myc* epitope (shown in red) was detected by immunofluorescence in non-permeabilized fibers treated with 100 nM insulin (20 min), 10 μM MCC950 (overnight), or both. Scale bar: 30 μm. (**b**) The bar graph shows the quantification of the effects presented in (**a**). Values represent the mean ± SEM (*n* = 4–5 independent experiments). Statistical analysis was performed by the Kruskal–Wallis test followed by Dunn’s post-hoc test. * *p* < 0.05, ** *p* < 0.01, and *** *p* < 0.001 vs. the basal NCD condition, and # *p* < 0.05 while ## *p* < 0.01 vs. the basal HFD condition.

In skeletal muscle, insulin promotes membrane trafficking of glucose transporter GLUT4 from GLUT4 storage vesicles to the sarcolemma and transverse tubular network, thereby facilitating glucose uptake [18]. Our previous reports showed that muscle-electroporation-based incorporation of the plasmidial vector GLUT4myc-eGFP, which encodes a chimeric protein, displayed stable expression of GLUT4myc-eGFP in murine FDB. This protein is functional and translocates to the plasma membrane in response to insulin incubation [19,20]. To evaluate the role of the inflammasome in GLUT4 translocation, we measured the fluorescent pattern of GLUT4myc-eGFP in isolated adult muscle fibers from FDB pre-incubated with MCC950, a potent and selective pharmacological inhibitor of NLRP3 inflammasome activity. Representative confocal images of adult fibers illustrated the effects of insulin on GLUT4myc-eGFP translocation (Figure 7a). As expected, 100 nM insulin promoted an increase in GLUT4myc-eGFP membrane translocation (3.4 ± 0.7-fold) compared to fibers in the basal condition isolated from NCD-fed mice (1.0 ± 0.1-fold). In contrast, this increase was not observed in HFD fibers, which reached similar values for the insulin incubation and basal condition (1.5 ± 0.3- and 1.1 ± 0.1-fold). On the other hand, pretreatment of fibers isolated from NCD- or HFD-fed mice with 10 μM MCC950 significantly increased the cell surface signal of GLUT4myc-eGFP by 4.6 ± 0.6- and 3.7 ± 0.7-fold. Interestingly, pretreatment with MCC950 plus insulin led to values of 3.7 ± 0.6- and 3.9 ± 0.5-fold in NCD- and HFD-fed mice, respectively (Figure 7b). Taken together, our results suggest that pharmacological inhibition of the NLRP3 inflammasome enhances GLUT4myc-eGFP translocation onto the cell surface of skeletal muscles from animals with insulin resistance, which could directly improve the glucose transport in this pathological condition.

## 3. Discussion

Chronic feeding with a high-fat diet overloads the metabolic machinery and alters the lipid and carbohydrate homeostasis. These factors are associated with the development of low-grade chronic inflammation and are related to insulin resistance [21]. In this study, we have reported for the first time the presence of active NLRP3 inflammasome complexes in adult skeletal muscle fibers from HFD-fed mice. Numerous studies have linked NLRP3 inflammasome complex activation with pathological conditions in the kidneys [22], heart [23], brain [24], gastrointestinal system [25], and hematopoietic tissue [26], among others. The NLRP3 inflammasome has also been described in cells directly involved in immune activity, such as neutrophils, macrophages, and dendritic cells [27]. In the latter cells, an association between the activity of the NLRP3 inflammasome complex with the innate immune response and pro-inflammatory effects in various autoimmune diseases has been well-established [28]. Mounting evidence supports the hypothesis that insulin resistance is caused by a chronic low-grade pro-inflammatory state, with increased circulatory levels of several pro-inflammatory cytokines, including IL-1β [29]. Our data were consistent with previous studies showing an elevation of plasma IL-1β levels in HFD-fed mice compared to NCD-fed animals [30]. Interestingly, activation of the NLRP3 inflammasome complex has also been described in insulin-sensitive tissues, for instance, in white adipose tissue and the liver [31,32], promoting the pro-inflammatory processes associated with metabolic disorders, such as insulin resistance, and other related illnesses, such as nonalcoholic fatty liver disease [33]. These processes have been linked to an increase in the activity of caspase-1 and, consequently, elevated protein levels of mature IL-1β [34]. To date, the presence of NLRP3 inflammasome complex components in muscle cells has been described in response to chronic pro-inflammatory stimuli in hereditary and autoimmune muscle pathologies [35,36,37,38].

Interestingly, our novel results showed a significant increase in the mRNA levels and protein content of NLRP3 inflammasome components in the skeletal muscle of insulin-resistant mice fed with an HFD for 8 weeks. These results support the idea that HFD muscle fibers experience an increased priming step, which is indispensable to NLRP3 inflammasome activation during IR. In addition, we have described the presence of the NLRP3 inflammasome in muscles isolated from HFD-fed animals that exhibit different metabolic characteristics, such as the FDB and soleus muscles. The FDB muscle shows a predominance of glycolytic-type fibers [39], whereas the soleus muscle presents mostly oxidative-type fibers [40]. At present, there are no descriptions of differences in the protein levels of NLRP3 inflammasome components according to skeletal muscle fiber typification, making this an area of interest for future work by our research group. McBride et al. described the effects of NLRP3 inflammasome activity in the skeletal muscle of 10- and 24-month-old mice, suggesting that NLRP3 inflammasome activity can be modulated by the skeletal muscle mass declining, and accordingly a decrease in the size of glycolytic fibers is associated with aging [41]. However, there is still not enough evidence to differentiate the activity of the NLRP3 inflammasome pathway according to the phenotypic and metabolic characteristics of muscle fibers.

The localization of an active NLRP3 inflammasome complex, mature IL-1β, and GSMD in the skeletal muscle place the muscle as a source of local inflammation mediated by IL-1β release. Interestingly, our results showed the localization of the components of the NLRP3 inflammasome complex in muscle fibers isolated from animals fed an HFD and NCD. However, there is no consensus regarding the effect exerted by the activity of the NLRP3 inflammasome directly on muscle tissue. So far, we have localized the NLRP3 inflammasome components within the isolated muscle fiber; this is a contribution to the initial understanding of chronic low-grade pro-inflammatory mechanisms associated with skeletal muscle tissue in an obesity context. Taken together, our results showed the presence and upregulation of NLRP3 inflammasome components in isolated muscle fibers during IR, supporting the idea of the skeletal muscle tissue as an active component of the inflammatory processes associated with obesity. On the other hand, it has been suggested that an increase in the protein levels of the NLRP3 inflammasome does not necessarily indicate an increase in its activity [42]. On this basis, we reported an increase in caspase-1 activity as a key marker of NLRP3 inflammasome function [43]. Recently, Boucher et al. suggested that caspase-1 activation is considered a necessary step for IL-1β maturation, and that it reflects NLRP3 inflammasome activity [44]. In addition, previous studies have suggested that caspase-1 activity is critical for GSDMD activation [14,45,46]. In a model of bone-marrow-derived dendritic cells KO for caspase-1, it was proposed that these cells lack the ability to release IL-1β into the extracellular milieu, and they fail to initiate pyroptosis upon stimulation with an NLRP3 inflammasome agonist [47]. Our results showed an increase in NLRP3 inflammasome activity in skeletal muscle tissue with altered insulin sensitivity, a relationship that was previously unknown.

IL-1β can negatively affect insulin signaling through a reduction in the levels of phosphorylated Akt, and also through serine phosphorylation of the insulin receptor by the autocrine and paracrine pathways [48,49]. In addition to the increase in NLRP3 inflammasome components, we detected a significant increase in mRNA and protein content for IL-1β in the skeletal muscle of insulin-resistant animals. This evidence supports a role of IL-1β in the pathogenesis of type 2 diabetes mellitus [50,51]. Among the mechanisms described to explain this alteration, it has been proposed that IL-1β can affect the tyrosine phosphorylation of the insulin receptor β subunit (IRβ) and insulin receptor substrate 1 (IRS-1) in pancreatic cell lines [52], and can also inhibit the phosphorylation of Akt and ERK1/2 induced by insulin in differentiated 3T3-L1 adipocytes [53]. Interestingly, IL-1β impairs both the glucose uptake in response to insulin and the expression of IRS-1 in C2C12 myotube cell lines [54]. De Roos et al. reported an increased expression of the IL-1RI (IL-1β receptor) in the skeletal muscle of mice fed with a high-fat diet [55]. Additionally, an influence of IL-1β secretion by skeletal muscle cells in pathological conditions such as sepsis has been proposed, where it mediates and promotes the development of muscle atrophy [56]. Based on the above, we propose that the increase in IL-1β mediated by skeletal muscle could promote IR in this tissue. In this work, we assessed the presence of IL-1β within the muscle fibers; however, future studies will allow us to elucidate whether the skeletal muscle releases IL-1β during IR-associated obesity. Additionally, we showed an increase in the protein levels of GSDMD in the skeletal muscle of HFD animals. GSDMD has been reported as a protein that mediates the release of IL-1β into the extracellular milieu [57]. This is due to the fact that IL-1β lacks the peptide signal that allows its secretion through conventional pathways, via the endoplasmic reticulum and Golgi apparatus [58]. The N-terminus of GSDMD is inserted and assembled in the plasma membrane, forming pore structures and triggering pyroptosis in macrophages [59]. Although the relationship of GSDMD with the development of pyroptosis suggests that its presence in the muscle cell should favor the activation of death pathways, Heilig et al. reported that IL-1β can be secreted even in sub-lytic states [57]. Future experiments will allow us to elucidate the role of GSDMD in insulin resistance and during glucose transport in the skeletal muscle.

MCC950, a specific inhibitor of NLRP3 inflammasome activation [60], has been recently used as a treatment for various NLRP3-related pathological models, including frontotemporal dementia [61], age-related metabolic syndrome [62], and obesity-induced insulin resistance [23,61]. Intraperitoneal administration of MCC950 improves glucose tolerance [23,62,63], insulin effects [61,63], and the insulin signaling response in skeletal muscle tissue [61]. Insulin-induced GLUT4 translocation is a critical step in this process [64]. However, MCC950′s effects on GLUT4 translocation in isolated muscle fibers have not been explored. Our results showed, for the first time, that MCC950 increases insulin-induced GLUT4 translocation in isolated skeletal muscle fibers from HFD mice. Previous reports have shown that MCC950 treatment improves insulin sensitivity and reduces circulating plasma insulin levels [61,65]. Considering that the model of isolated muscle fibers excludes the influence of other cells, these results suggest that, during IR, the activation of the NLRP3 inflammasome expressed within the skeletal muscle may disrupt the insulin response through an autocrine or paracrine action of the cytokines involved (possibly through IL-1β). Interestingly, our data showed that MCC950 increases GLUT4 translocation in the absence of insulin, in both NCD and HFD muscle fibers. This suggests that the NLRP3 inflammasome might play a physiological role in glucose metabolism, as a regulator of GLUT4 trafficking in skeletal muscle.

## 4. Materials and Methods

### 4.1. Animals

Male C57BL/6J mice were obtained from the Animal Facility at the Universidad de Chile. The mice were maintained in a controlled environment; the room temperature was constant at 21 °C and a 12:12 h light-dark cycle was utilized. At 21 days old, the mice were divided into two groups: the normal control diet (NCD) group received a diet containing (w/w) 10% fat, 20% protein, and 70% carbohydrate, and the high-fat diet (HFD) group was given a diet containing (w/w) 60% fat, 20% protein, and 20% carbohydrate (D12492, Research Diets, New Brunswick, NJ, USA). The animals were sacrificed after 8 weeks of treatment. The Bioethics Committee of the Faculty of Medicine approved all animal procedures performed in this work.

### 4.2. Analysis of Plasma Parameters

The mice were fasted for 4–6 h and an intraperitoneal glucose tolerance (IGTT) test was performed by administration of a glucose bolus of 2 g/kg. At 0, 15, 30, 60, and 120 min, blood was collected from the tip of the tail of each mouse, and blood glucose was measured with a One Touch II glucose meter (Lifescan, Mountain View, CA). Fasting insulin and plasma IL-1β were measured with a rat/mouse insulin ELISA kit (Merck, Darmstadt, Germany) and a mouse IL-1β ELISA kit (R&D Systems, Minneapolis, MN, USA), respectively. Briefly, the mice were anesthetized and terminal blood samples were collected by cardiac puncture in tubes. The samples were centrifuged at 5000× *g* for 15 min at 4 °C to separate the plasma fraction, and then the samples were aliquoted and immediately frozen at −80 °C until further processing.

### 4.3. Adult Skeletal Muscle Fiber Cultures

The procedure was described previously [20]. Briefly, isolated fibers from the FDB muscle were obtained by enzymatic digestion of the whole muscle for 90 min at 37 ºC with collagenase type 4 (Worthington, Lakewood, NJ, USA). After this, the muscle was mechanically dissociated by passage through fire-polished Pasteur pipettes. Isolated fibers were seeded in ECM-coated coverslips, and maintained in Dulbecco’s Modified Eagle Medium (Invitrogen, Carlsbad, CA, USA) supplemented with 10% horse serum (Invitrogen, Carlsbad, CA, USA) in an incubator controlled at 37 °C, 95% humidity, and 5% CO_2_. The fibers were used no longer than 8 h after isolation.

### 4.4. Western Blot Analysis

The FDB or soleus muscles from mice were homogenized by sonication in cold RIPA lysis buffer, containing 140 mM NaCl, 1 mM EDTA, 1 mM EGTA, 1 mM BAPTA, 20 mM Tris-HCl, pH 7.5, 1% Triton X-100, and Protease Inhibitor Cocktail (Roche Diagnostics, Germany), as described previously [20]. The samples were loaded onto a 12% SDS-polyacrylamide gel and then transferred to a polyvinylidene difluoride membrane (Millipore, Burlington, MA, USA). The primary antibodies used were: anti-NLRP3 (1:500) from R&D Systems (MAB7578, Minneapolis, MN, USA), anti-IL-1β (1:500; SC-32294), anti-caspase-1 (1:500; SC-56036), anti-ASC (1:1000; SC514414), and anti-GSDMD (1:500; SC-393581) from Santa Cruz Biotechnology (Dallas, TX, USA), and anti-GADPH (1:20000) from Sigma-Aldrich (G9545; St. Louis, MO, USA). After washing, the membranes were incubated for 1.5 h with secondary anti-rabbit, anti-rat, or anti-mouse antibodies, as appropriate (Sigma-Aldrich, St. Louis, MO, USA). Western blotting detection reagents (LumiFlash™ Infinity Chemiluminescent Substrate, Taipei, Taiwan) were used following the manufacturer’s instructions, and chemiluminescence was detected using a ChemiDoc Imaging System from Bio-Rad (Hercules, CA, USA). The intensity of the bands was quantified by densitometry, with the use of ImageJ software version 1.44p (NIH, Bethesda, MD, USA).

### 4.5. Immunofluorescence Assays

Skeletal muscle fibers were plated on 35 mm coverslips, where they were washed with PBS and fixed by incubation for 10 min at room temperature with PBS containing 4% paraformaldehyde (Electron Microscopy Science, Hatfield, PA, USA). Next, the fibers were rinsed with PBS, permeabilized with a solution containing 0.1% Triton X-100 in PBS, rinsed with PBS, and blocked for 1 h with PBS-1% BSA at room temperature. Monoclonal antibodies against NLRP3 (1:50; MAB7578; R&D Systems, Minneapolis, MN, USA), IL-1β (SC-32294), ASC (SC514414), caspase-1 (SC-56036), and GSDMD (SC-393581) (1:50; Santa Cruz Biotechnology, Dallas, TX, USA) were used to detect these proteins. Fibers were washed and then incubated for 1 h with Alexa Fluor-488 anti-mouse and Alexa Fluor-546 anti-rat antibodies, respectively (1/250 dilution, Molecular Probes, Invitrogen, Carlsbad, CA, USA). DAPI was used to identify nuclei in skeletal muscle fibers. Samples were treated with Dako anti-fading reagent (Dako North America, CA, USA) and stored at 4 ºC until use.

### 4.6. Quantitative Real-Time PCR (qRT-PCR)

Total RNA was isolated from the FDB muscle using Trizol (Invitrogen, Corp., Carlsbad, CA, USA), according to the manufacturer’s protocol. Reverse transcription to cDNA and qPCR amplification were performed according to the procedures described previously [7]. Expression values were normalized to housekeeping gene Rplp0 (sense: CTCCAAGCAGATGCAGCAGA; antisense: ATAGCCTTGCGCATCATGGT) and reported in units of 2−ΔΔCt ± SEM. The NLRP3, ASC, Pro-CASP1, IL-1β, and GSDMD primers were: NLRP3 (sense: TGCAACCTCCAGAAACTGTG; antisense: AGAACCAATGCGAGATCCTG) sequence), ASC (sense: ACAGCCAGAACAGGACACTTT; antisense: CTCCGTCCACTTCTGTGACC sequence), IL-1β (sense: GCAACTGTTCCTGAACTCACCT; antisense: ATCTTTTGGGGTCCGTCAACT) sequence), Pro-CASP1 (sense: GCCGTGGAGAGAAACAAGGA; antisense: TCCAAGTCACAAGACCAGGC) sequence) and GSDMD (sense: TGAAGCACGTCTTGGAACAG; antisense: TCTTTTCATCCCAGCAGTCC sequence).

### 4.7. Caspase-1 Fluorometric Assay

The caspase-1 activity was assayed using a caspase-1 fluorometric assay kit (BioVision, Inc., Milpitas, CA, USA), following the manufacturer’s protocols. Briefly, gastrocnemius muscle was homogenized using the kit lysis buffer (1:4), and a reaction buffer was used with DTT and the substrate YVAD-AFC. The measurement was carried out in a kinetic way at 37 °C. Plates were read at an excitation of 400 nm and emission of 505 nm, using a Sinergy fluorescence plate reader (BioTek Instruments, Winooski, VT, USA). The recordings were performed in 15 s intervals during a period of 3 min, after pre-incubation of 1 min. Activity was expressed as fluorescence min^−1^·mg^−1^ change.

### 4.8. GLUT4myc-eGFP Electroporation in FDB Muscle

Plasmid injection and electroporation were performed as described previously [19,20]. Briefly, FDB muscles were injected with 2 mg/mL hyaluronidase, and 1 h later the muscles were injected again with a total of 20 μg of GLUT4*myc*-eGFP. A pair of electrodes were placed under the skin, and oriented parallel to each other and perpendicular to the long axis of the foot. The electroporation was performed using ~100 V/cm, 20 pulses, 20 ms in duration each, at 1 Hz frequency, which was generated using a Grass stimulator (Grass S48; W. Warwick, RI, USA). The expression of GLUT4*myc*-eGFP was assayed 10 days after transfection.

### 4.9. GLUT4myc-eGFP Translocation Assays

Fibers electroporated with GLUT4*myc*-eGFP chimera plasmids were seeded on glass coverslips, which was followed by serum deprivation for 3 h. Cells were pre-incubated overnight with 10 μM MCC950 (Calbiochem, Sigma-Aldrich, Burlington, MA, USA), 100 nM insulin (Actrapid, Novo-Nordisk, Denmark), or both. Labeling of surface GLUT4*myc* in nonpermeabilized cells was performed as described [19,20]. Briefly, after washing with PBS, the fibers were fixed by incubation for 10 min at room temperature with PBS containing 4% paraformaldehyde (Electron Microscopy Science, Hatfield, PA, USA). Next, the skeletal muscle fibers were rinsed with PBS and blocked for 1 h with PBS-1% BSA at room temperature. GLUT4*myc*-eGFP levels at the cell surface were detected with the monoclonal anti-*myc* antibody (1/100 dilution, SC-40; Santa Cruz Biotechnology, Santa Cruz, CA, USA), followed by treatment with a secondary antibody conjugated with AlexaFluor 635 (1/500 dilution, Molecular Probes, Invitrogen, Carlsbad, CA, USA). Images were obtained using a C2+ confocal microscope system (Nikon Instruments Inc., Minato, Tokyo, Japan). GLUT4*myc*-eGFP translocation analysis was performed as described in [66].

### 4.10. Statistical Analysis

Data are presented as the mean ± SEM. Significant differences between and within multiple groups were examined using Kruskal–Wallis for repeated measures, followed by the multiple comparison test. Student’s *t*-test was used to detect significant differences between the two groups. *p* < 0.05 was considered statistically significant. GraphPad Prism 9 software (GraphPad Software Inc, San Diego, CA) was used for statistical analysis of the data and plot construction.

## 5. Conclusions

As of yet, the molecular mechanisms underlying the development of IR have not been fully described. Due to the deleterious consequences of this pathological condition, understanding of the pathways that favor its development is of great interest. It has been described that NLRP3 inflammasome activity affects intracellular insulin signaling and glucose homeostasis in sensitive tissues [9]. Here, we located the main components of the NLRP3 complex in skeletal muscle, and concluded that the activation of this inflammasome is linked to increments in circulating and skeletal-muscle-associated IL-1β during insulin resistance. The inhibition of NLRP3 inflammasome activity promotes GLUT4 translocation, improving insulin sensitivity in isolated skeletal fibers from obese mice.

## Supplementary Materials

The following are available online at https://www.mdpi.com/article/10.3390/ijms221910212/s1.
