# Supplementary material for: Activation of the NLRP3 Inflammasome Increases the IL-1β Level and Decreases GLUT4 Translocation in Skeletal Muscle during Insulin Resistance
Source: Int J Mol Sci. 2021 Sep 23;22(19):10212. doi: 10.3390/ijms221910212 (PMC8508423; doi:10.3390/ijms221910212)
Supplement: Supplementary file 1 [file ijms-22-10212-s001.zip › ijms-1356974-supplementary.pdf]

International Journal of
Molecular Sciences

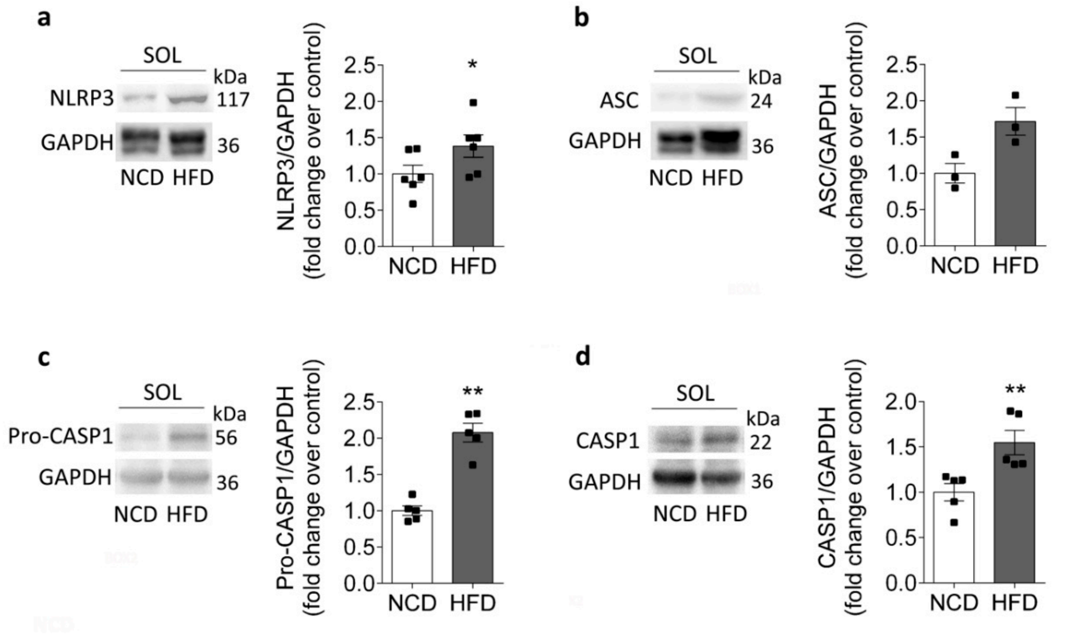

Supplementary Materials Figure S1. Elevated protein content of the NLRP3 inflammasome in the soleus muscle from HFD- compared to NCD-fed mice. Representative Western blot and quantification showing (**a**) NLRP3, (**b**) ASC, (**c**) pro-caspase-1, and (**d**) caspase-1 protein content. The measures were performed with homogenates prepared from the soleus muscle isolated from NCD- or HFD-fed mice after 8 weeks on the diets. GAPDH was used as a housekeeping loading control. Each lane was loaded with 60 µg of protein. Values represent the mean ± SEM (n = 3–6). * $p < 0.05$ and ** $p < 0.01$, determined by Student's t-test. NCD, normal control diet; HFD, high-fat diet.
